# Impact factors for quantifying country-level terrestrial biodiversity intactness footprints (IBIF)

**DOI:** 10.1038/s41597-025-05946-1

**Published:** 2025-10-21

**Authors:** Aafke M. Schipper, Martijn van der Marel, Michel Bakkenes, Paul Giesen, Mark M. P. van Oorschot, Harry C. Wilting, Mark A. J. Huijbregts, Alexandra Marques

**Affiliations:** 1 PBL Netherlands Environmental Assessment Agency, The Hague, The Netherlands; 2 Radboud Institute for Biological and Environmental Sciences (RIBES), Radboud University, Nijmegen, The Netherlands

**Keywords:** Environmental impact, Biodiversity

## Abstract

There is an increasing demand for consistent methods and tools to quantify biodiversity footprints: the magnitude of biodiversity loss associated with all direct and indirect impacts associated with a given human activity or economic actor. Here, we present the intactness-based biodiversity impact factors (IBIF) dataset: a consistent set of country-level impact factors that can be used to attribute losses in local terrestrial biodiversity intactness to emissions and resource use associated with production or consumption in a given country. We used the GLOBIO biodiversity model and its mean species abundance (MSA) metric to obtain these impact factors for 234 countries and five environmental pressures: CO_2_ emissions, NH_3_ emissions, NO_x_ emissions, land use (urban land, cropland, pasture, forest plantations and mines) and roads. IBIF includes impact factors for vascular plants, warm-blooded vertebrates (birds & mammals) and both species groups combined. The dataset can be used to quantify the biodiversity footprints of current products, industrial sectors or consumers, in support of policy- and decision-making aimed at halting or reversing biodiversity loss.

## Background & Summary

Halting the ongoing loss of biodiversity is one of the major global challenges of the 21st century^1–3^. Despite numerous international scientific studies and policy agreements confirming that the conservation of biological diversity is a global priority, biodiversity trends continue mostly downward^4^. As it is increasingly acknowledged that the conservation of biodiversity is a shared responsibility of public, private and non-profit actors^5,6^, there is a growing demand for science-based, quantitative approaches and indicators for evaluating the performance of a given actor in terms of its impacts on biodiversity^7,8^. In the current Kunming-Montreal Global Biodiversity Framework, this demand has been formalized with Target 15 on enabling and encouraging business and finance actors to assess and disclose their impacts on biodiversity along their operations, including impacts embedded in their supply chains^5^.

Biodiversity footprints aim at a comprehensive quantification of the biodiversity impacts of specific activities of a given actor, including not only the direct but also the indirect impacts, the latter caused by upstream supply chains^7,9^. Quantifying biodiversity footprints requires an inventory of the relevant environmental interventions or pressures associated with a given activity, such as the amount of greenhouse gasses emitted or the amount of land needed. The numbers resulting from this inventory are then multiplied with impact factors or loss factors, also known as characterization factors in the context of Life Cycle Assessment (LCA). These impact factors represent the amount of biodiversity loss associated with a unit of pressure (i.e., emission or resource use)^9,10^. Biodiversity loss factors are commonly based on the potentially disappeared fraction (PDF) of species, which is a common metric in widely used life cycle impact assessment (LCIA) methodologies, such as ReCiPe and LC-IMPACT^10,11^. PDF ranges from 0 to 1, where 0 denotes that all original species are extant and 1 that all original species are threatened with extinction. The mean species abundance (MSA) metric provides an alternative measure of anthropogenic impacts on biodiversity, representing average declines in the local abundance of species. Being based on abundance, MSA is more sensitive to changes in ecological communities than PDF, which offers a potential advantage if the aim is to track biodiversity losses and gains^12^. However, in spite of a few first efforts to quantify MSA-based biodiversity footprints^13–17^, a consistent set of MSA-based biodiversity impact factors is yet lacking.

**Fig. 1:**
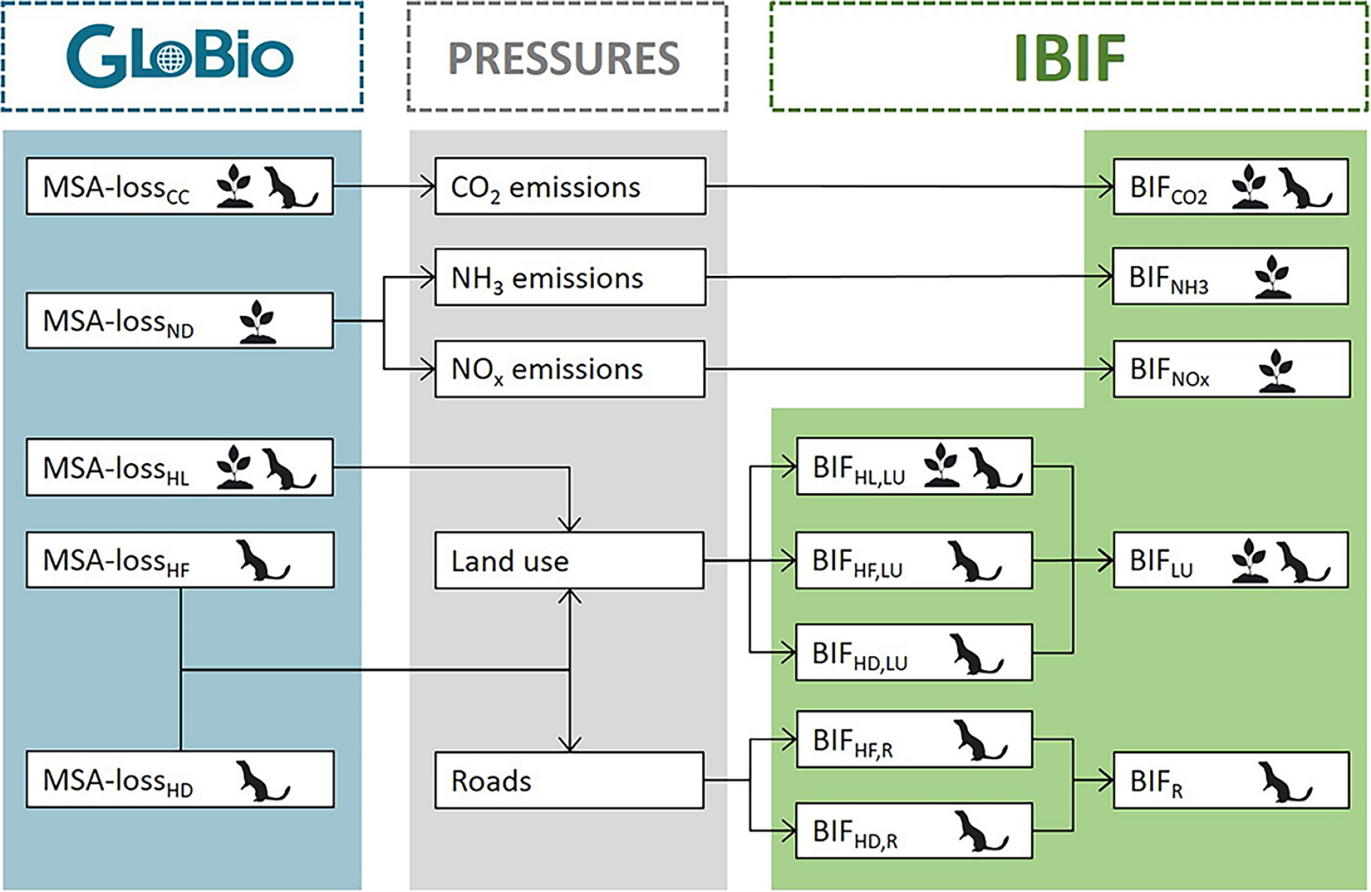
Schematic representation of the workflow followed to retrieve the IBIF dataset from the GLOBIO model. GLOBIO is used to quantify losses in local community intactness of plants and/or warm-blooded vertebrates (birds & mammals), as expressed by the mean species abundance (MSA) metric, due to climate change (CC), nitrogen deposition (ND), habitat loss (HL), habitat fragmentation (HF) and habitat disturbance (HD). The losses in MSA are then allocated to the underlying pressures (CO_2_ emissions, NH_3_ emissions, NO_x_ emissions, land use, and roads) for establishing biodiversity impact factors (BIFs). IBIF contains BIFs for each of these five pressures as well as BIFs for the direct drivers underlying the impacts of land use and roads (habitat loss, habitat fragmentation, habitat disturbance).

Here, we present the Intactness-based Biodiversity Impact Factors (IBIF) dataset, a novel and consistent set of country-level biodiversity impact factors representative of declines in local terrestrial biodiversity intactness as represented by the MSA metric. To establish IBIF, we first employed the global biodiversity model GLOBIO to quantify losses in MSA relative to undisturbed conditions due to climate change, atmospheric nitrogen deposition, habitat loss, habitat fragmentation and habitat disturbance. We then attributed these losses to the underlying pressures (CO_2_ emissions, NH_3_ emissions, NO_x_ emissions, land use and roads) for each of 234 countries, in order to quantify the loss in terrestrial biodiversity intactness associated with a unit of each pressure generated in the country of concern (Fig. 1). The resulting biodiversity impact factors can be combined with life cycle inventory (LCI) data or environmentally extended multi-regional input-output (EE-MRIO) models to quantify the biodiversity footprints of products, sectors or consumers, which in turn can be used to support policy- and decision-makers in developing adequate responses to prevent or reduce further biodiversity loss. For example, they can be used in the (national) implementation of Target 15 of the Kunming-Montreal Global Biodiversity Framework, as well as in the implementation of the European Corporate Sustainability Reporting Directive (CSRD).

## Methods

### GLOBIO 4 model

To establish IBIF, we first quantified worldwide human-induced losses in local terrestrial biodiversity intactness with the global biodiversity model GLOBIO^18,19^. GLOBIO quantifies local biodiversity intactness using the mean species abundance (MSA) metric, which is a dimensionless metric between 0 and 1 where 1 denotes a species assemblage that is fully intact while 0 indicates that all species of the original assemblage are extirpated. The current version of GLOBIO (version 4) estimates losses in MSA for two taxonomic groups, namely vascular plants and warm-blooded vertebrates (birds & mammals), at a spatial resolution of 10 arc-seconds (approximately 300 m at the equator)^19^. GLOBIO quantifies MSA losses of plants as a function of three direct drivers (i.e., human activities or anthropogenic environmental changes that directly affect organisms): anthropogenic climate change, atmospheric nitrogen deposition and habitat loss. MSA losses for warm-blooded vertebrates are quantified as a function of five direct drivers: anthropogenic climate change, habitat loss, habitat fragmentation, habitat disturbance, and direct exploitation (hunting)^19^. Habitat loss, in turn, is caused by land use, while habitat fragmentation and habitat disturbance are caused by both land use and roads.

At the core of the GLOBIO model is a set of empirical impact relationships that quantify MSA as a function of each direct driver, parameterized through meta-analytical beta-regression analyses of global datasets^19^. GLOBIO uses these impact relationships to estimate MSA values for each direct driver and each 10 arc-seconds grid cell. The MSA maps for the individual direct drivers are then combined in order to estimate the overall MSA per grid cell. If the impact of a given direct driver is assumed to be subordinate to the impact of another direct driver, the impact due to the subordinate driver is excluded from the calculation of the combined MSA for that grid cell^19^. For example, it is assumed that atmospheric nitrogen deposition does not have additional impacts on plant communities in agricultural land, which is typically fertilized. Hence, in agricultural lands, the MSA of plants is calculated based on the impacts of habitat loss and climate change only^19^. Upon exclusion of the subordinate direct drivers, the overall MSA is calculated as:

1 $${\text{MSA}}_{g,i}={\prod }_{x}{{MSA}}_{x,g,i}$$

where MSA_g,i_ is the MSA of species group *g* in grid cell *i*, and MSA_x,g,i_ is the MSA of species group *g* due to direct driver *x* in grid cell *i*. After having calculated the overall MSA, GLOBIO estimates the contribution of each direct driver to the total loss in MSA (i.e., 1-MSA) in each grid cell as^19^:

2 $${\text{MSA} \mbox{-} \text{loss}}_{x,g,i}=\frac{1-{MSA}_{x,g,i}}{{\sum }_{x}(1-{MSA}_{x,g,i})}\cdot (1-{MSA}_{g,i})$$

where MSA-loss_*x,g,i*_ is the loss in MSA due to direct driver *x* for species group *g* in grid cell *i*. Thus, the loss in MSA in a given grid cell attributed to a given direct driver *x* is conditional on the impacts of the other direct drivers that contribute to the total loss in MSA in that same grid cell (i.e., *1 – MSA*_*g,i*_).

### GLOBIO settings

For creating IBIF, we used GLOBIO version 4 as described by Schipper *et al*.^19^ with a few updates and modifications (version v4.3.1). First, we excluded the impacts of direct exploitation, because of difficulties in attributing impacts of hunting (especially illegal and subsistence hunting) to economic activities. Second, we revised the impact relationship for habitat loss due to land use, in view of the small sample sizes for some of the land use intensity levels. To that end, we pooled the data across intensity levels and refitted the impact relationship based on four aggregated land-use types (urban land, cropland, pasture and forest plantations; see Table 1). Further, we added mines as a separate land-use category, in order to be able to establish impact factors specific to mining. In the implementation in GLOBIO, we assumed a complete loss of biodiversity at the extraction sites themselves (so, MSA = 0 or MSA-loss = 1 for habitat loss by mines). We assumed that mines also cause habitat disturbance, which we quantified based on the same MSA loss relationship as used for disturbance by roads, in absence of a mining-specific relationship. While we acknowledge that this is a simplistic and uncertain approximation, we preferred this approach over just neglecting disturbing effects beyond the extraction site itself, as it is known that mining has considerable off-site effects on surrounding habitat^20,21^. We further assumed the impacts of disturbance to be restricted to an impact zone of 5 km from mines and roads^22^. In case of overlapping impact zones due to multiple infrastructure elements, we assign the biggest impact (so the impact due to the closest mine or road) to the grid cell of concern.

**Table 1:** MSA impact relationships in GLOBIO used for establishing IBIF.

Direct driver	Driver variable (unit)	Plants			Birds & mammals	
		*Impact relationship*	*n*	*Impact relationship*		*n*
Climate change	Global mean temperature increase (GMTI; °C)	MSA = 1/(1 + e^−2.87 + 0.467 · GMTI^)	135	MSA = 1/(1 + e^−3.21 + 0.362 · GMTI^)		141
Nitrogen deposition	Nitrogen deposition (ND; kg · ha^−1^ · yr^−1^)	MSA = 1/(1 + e^−2.19 + 0.743 · log(ND)^)	89	NA		NA
Habitat loss	Cropland	MSA = 1/(1 + e^1.44^)	9	MSA = 1/(1 + e^0.296^)		17
	Pasture	MSA = 1/(1 + e^1.11^)	14	MSA = 1/(1 + e^0.135^)		14
	Plantation forest	MSA = 1/(1 + e^0.920^)	15	MSA = 1/(1 + e^−0.212^)		30
	Urban land	MSA = 1/(1 + e^1.10^)	3	MSA = 1/(1 + e^1.15^)		5
Habitat fragmentation	Patch size (PS) of semi-natural habitat (ha)	NA	NA	MSA = 1/(1 + e^0.774 - 0.594 · log(PS)^)		39
Habitat disturbance	Distance (D) from road or mine (m)	NA	NA	MSA = 1/(1 + e^1.77 - 1.39 · log(D)^)		204

Impact relationships were fitted through weighted beta regression modelling, with the square root of the number of species per observation as weight^19^. n represents the number of MSA values underlying each impact relationship.

### GLOBIO input data and model runs

Running GLOBIO requires input data on climate change (global mean temperature increase since pre-industrial times; °C), atmospheric nitrogen deposition (kg · ha^−1^ · yr^−1^), land use, and the presence of roads. Based on land use (urban land, cropland and pasture) and roads, GLOBIO calculates the sizes of remaining natural habitat patches, as input to estimate fragmentation impacts (Table 1). For climate change, we used a global mean temperature increase of 1.26 °C, which corresponds with the level of global warming in 2020 compared to pre-industrial times according to the MAGICC6 climate model included in the IMAGE model framework^23^. We used a global map of total atmospheric nitrogen deposition (kg · ha^−1^ · yr^−1^) representative of the year 2020 from Zhu *et al*.^24^, who generated global N deposition grids at a resolution of 0.125° × 0.125° for 2008–2020^25^. We compiled a land-use map by combining the 10 arc-seconds ESA CCI land‐cover map for 2020^26^, which already includes urban land and cropland, with country-level estimates on the areas of pasture and plantation forest, which we allocated to the grid cell level using the GLOBIO land allocation routine (see Schipper *et al*.^19^ and Meijer^27^ for details on the allocation procedure). We obtained the country-level estimates of pasture and plantation forest areas for the year 2020 from the land-use data provided by FAOSTAT (https://www.fao.org/faostat/en/#data/RL). For pasture, we used the sum of the areas of permanent and temporary meadows and pastures. For plantation forest, we used the smaller of two estimates at the country level: i) the total area of planted forest (defined as forest predominantly composed of trees established through planting and/or deliberate seeding) or ii) the total area of forest minus the area of forest for biodiversity restoration. We included the second estimate because the category ‘planted forest’ may include forest specifically planted for biodiversity restoration, which we consider not sufficiently representative of production forest plantations. We combined the land-use map resulting from the allocation procedure with the global mining site map compiled by Maus *et al*.^28^, which contains 44,929 mining polygons across the globe^29^. We obtained road data from the Global Road Inventory Project (GRIP) database^30^, which contains globally consistent road data distinguishing five road types^31^. Similar to Schipper *et al*.^19^, we assumed that impacts due to roads are exclusively caused by highways, primary roads and secondary roads (road types 1–3 in GRIP), excluding road types 4 and 5 because minor roads are much less disturbing to wildlife^32^. To quantify separate BIFs for habitat disturbance by land use (mines) and roads, we attributed habitat disturbance effects in a grid cell to the closest mine or road. To tease apart the habitat fragmentation effects from land use and roads, which are aggregated within GLOBIO, we performed three GLOBIO model runs: one default run, one run excluding roads, and one run excluding land use. Based on the results of the default run (fragmentation due to land use and roads combined) and the two extra runs, we attributed the combined fragmentation impact (full run) to land use and roads proportional to their individual fragmentation impacts (extra runs).

### BIF calculations

#### Rationale and scope

Fundamental to quantifying biodiversity footprints is the need to account for the total impact on biodiversity due to a given activity or economic actor. This, in turn, requires consideration of both the total area across which the impact takes place and the duration of the impact^11^. For this reason, we aimed to design the impact factors in IBIF such that they allow for quantifying the area- and time-integrated biodiversity loss due to the pressures associated with a given activity or economic actor as represented in life cycle inventory (LCI) or environmentally extended multi-regional input output (EEMRIO) data. BIFs for the emissions of CO_2_, NH_3_ and NO_x_ are therefore expressed in terms of MSA-loss integrated over area and time per kg of substance emitted in the country of concern (MSA-loss · km^2^ · yr · kg^−1^). For land use, we provide BIFs representing the area-integrated MSA-loss per unit of land area occupied (MSA-loss · km^2^ · km^−2^), leaving out the temporal dimension because the duration of use of a given unit of land is typically represented or implied by LCI or EEMRIO data^11^. For roads, we provide BIFs representing the area-integrated MSA-loss per unit of road length, as this is in line with how impacts are modelled within GLOBIO. We explain in the usage notes section how the BIFs for roads can be converted in order to harmonize them with the information available in LCI or EEMRIO databases.

#### CO_2_ emissions

Because CO_2_ emissions in a given country affect the global climate, we calculated BIFs for CO_2_ emissions (both plants and warm-blooded vertebrates) based on the cumulative global MSA loss due to climate change combined with the absolute global temperature change potential of CO_2_, as:

3 $${BIF}_{{CO}_{2},g}={IAGTP}_{{CO}_{2}}\cdot \frac{\sum _{i}{\text{MSA} \mbox{-} \text{loss}}_{CC,g,i}\cdot {A}_{i}}{GMTI}$$

where ${{BIF}}_{{{CO}}_{2},g}$ represents the biodiversity impact factor of CO_2_ (or CO_2_ equivalents) for species group *g* (plants or warm-blooded vertebrates; MSA-loss · km^2^ · yr · kg^−1^ CO_2_-eq), ${{IAGTP}}_{{{CO}}_{2}}$ represents the time-integrated absolute global temperature change potential of CO_2_ (°C · yr · kg^−1^), *MSA-loss*_*CC,g,i*_ represents the MSA loss of species group *g* due to climate change in grid cell *i*, as calculated with Eq. 2, *A*_*i*_ is the area of grid cell *i* (km^2^), and GMTI is the global mean temperature increase since preindustrial times (°C). Following the IPCC, we used a time horizon of 100 years, corresponding with an ${{IAGTP}}_{{{CO}}_{2}}$ of 47.6 ∙ 10^-15^ °C · yr · kg^−1^ CO_2_^33^. For GMTI, we used the same global mean temperature increase from preindustrial times to 2020 as used as input in the GLOBIO runs (i.e., 1.26 °C), to ensure consistency.

#### Nitrogen emissions

To quantify BIFs for the emissions of NH_3_ and NO_x_ (plants only), we attributed plant MSA losses due to the deposition of nitrogen to the emissions of NH_3_ and NO_x_ in the respective source countries, as

4 $${BIF}_{N,s,j}=\,\sum _{k}\frac{{N}_{s,j,k}}{{N}_{s,j}}\cdot \frac{\sum _{i,k}{\text{MSA} \mbox{-} \text{loss}}_{ND,i,k}\cdot {A}_{i,k}}{\sum _{i}{ND}_{i,k}}\cdot {NICF}_{s}$$

where *BIF*_*N,s,j*_ represents the biodiversity impact factor for nitrogen substance *s* (NH_3_ or NO_x_) for emitting country *j* (MSA-loss · km^2^ · yr · kg^−1^), *N*_*s,j,k*_ represents how much of nitrogen substance *s* emitted in country *j* ends up in receiving country *k* (kg · yr^−1^), *N*_*s,j*_ represents the total amount of nitrogen substance *s* emitted from country *j* (kg · yr^−1^), *MSA-loss*_*ND,i,k*_ represents the MSA loss due to the total nitrogen deposition in grid cell *i* in receiving country *k* (as calculated with Eq. 2), A_*i,k*_ is the area of grid cell *i* in receiving country *k* (km^2^), *ND*_*i,k*_ is the total amount of nitrogen deposited in grid cell *i* in receiving country *k* (kg · yr^−1^), and NICF_s_ is the substance-specific nitrogen-ion conversion factor needed to convert the amount (kg) of nitrogen deposited to the amount (kg) of either NO_x_ or kg of NH_3_ emitted (dimensionless). To link the deposition in the receiving countries to the emitting countries, as represented by the factor $\frac{{N}_{s,j,k}}{{N}_{s,j}},$ we used so-called source-receptor matrices (SRMs) from the global atmospheric chemical transport model TM5-FASST^34^. TM5-FASST simulates the transport of air pollutants based on 56 world regions, and the SRMs define, for each pollutant, how 1 kg of emission is distributed over the receiving regions. We used SRMs specific to NH_3_ and NO_x_ to obtain BIFs specific to each substance. We obtained the total nitrogen deposited in each grid cell (*ND*_*i,k*_) from the same layer used as input to the GLOBIO runs, to ensure consistency. Finally, we quantified the nitrogen-ion conversion factors based on the molecular weights of N, H and O and assuming that, under photostationary conditions, NO_x_ consists of 90% NO_2_ and 10% NO^35^. This resulted in conversion factors of 14/17 = 0.82 for NH_3_ and 0.90 · 14/46 + 0.10 · 14/30 = 0.32 for NO_x_. As countries are nested within TM5-FASST regions, countries within the same TM5-FASST region have the same BIF for either NH_3_ or NO_x_.

#### Land use (habitat loss, habitat fragmentation, habitat disturbance)

To quantify BIFs for land use (both plants and warm-blooded vertebrates), we divided the cumulative loss of MSA due to each land-use type in a given country by the total area of that land-use type within that country, as:

5 $${BIF}_{LU,l,g,j}=\,\frac{\sum _{i}{\text{MSA} \mbox{-} \text{loss}}_{LU,l,g,i,j}\cdot {A}_{i,j}}{\sum _{i}{A}_{LU,l,i,j}}$$

where *BIF*_*LU,l,g,j*_ represents the biodiversity impact factor for land-use (LU) type *l* for species group *g* in country *j* (MSA-loss · km^2^ · km^−2^), *MSA-loss*_*LU,l,g,j,i*_ represents the MSA loss due to land-use type *l* of species group *g* in grid cell *i* in country *j* (as calculated with Eq. 1), A_*i,j*_ is the area of grid cell *i* in country *j* (km^2^), and *A*_*LU,l,j,i*_ is the area of grid cell *i* used for land-use type *l* in country *j* (km^2^). We identified the land-use grid cells based on the same land-use map that was used as input to the GLOBIO runs, to ensure consistency. We performed the BIF calculations separately for each of the three direct drivers associated with land use (habitat loss, habitat fragmentation and habitat disturbance). In line with the set-up and parameterization of the GLOBIO model, we quantified BIFs for habitat loss for five land-use types (urban land, cropland, pasture, forest plantation, and mines), BIFs for habitat fragmentation for three land-use types (urban land, cropland and pasture) and a BIF for habitat disturbance for one land-use type (mines). In line with the current version of GLOBIO, habitat fragmentation and habitat disturbance are assumed to apply only to warm-blooded vertebrates (Fig. 1, Table 1).

#### Roads (habitat fragmentation, habitat disturbance)

To quantify BIFs for roads (warm-blooded vertebrates only), we divided the cumulative vertebrate MSA loss due to roads in a given country by the total length of the roads within that country, as:

6 $${BIF}_{R,j}=\,\frac{\sum _{i}{\text{MSA} \mbox{-} \text{loss}}_{R,i,j}\cdot {A}_{i,j}}{{RL}_{j}}$$

where *BIF*_*R,j*_ represents the biodiversity impact factor for roads in country *j* (MSA-loss · km^2^ · km^−1^), *MSA-loss*_*R,i,j*_ represents the MSA loss due to roads in grid cell *i* in country *j* (as calculated with Eq. 2), A_*i,j*_ is the area of grid cell *i* in country *j* (km^2^), and *RL*_*j*_ is the total length of roads in country *j* (km). We obtained the total road length by summing the length of road types 1–3 per country, as obtained from the same global road network map used as input to run GLOBIO^30^. We performed these calculations separately for each of the two direct drivers associated with roads (habitat fragmentation and habitat disturbance).

## Data Records

**Fig. 2:**
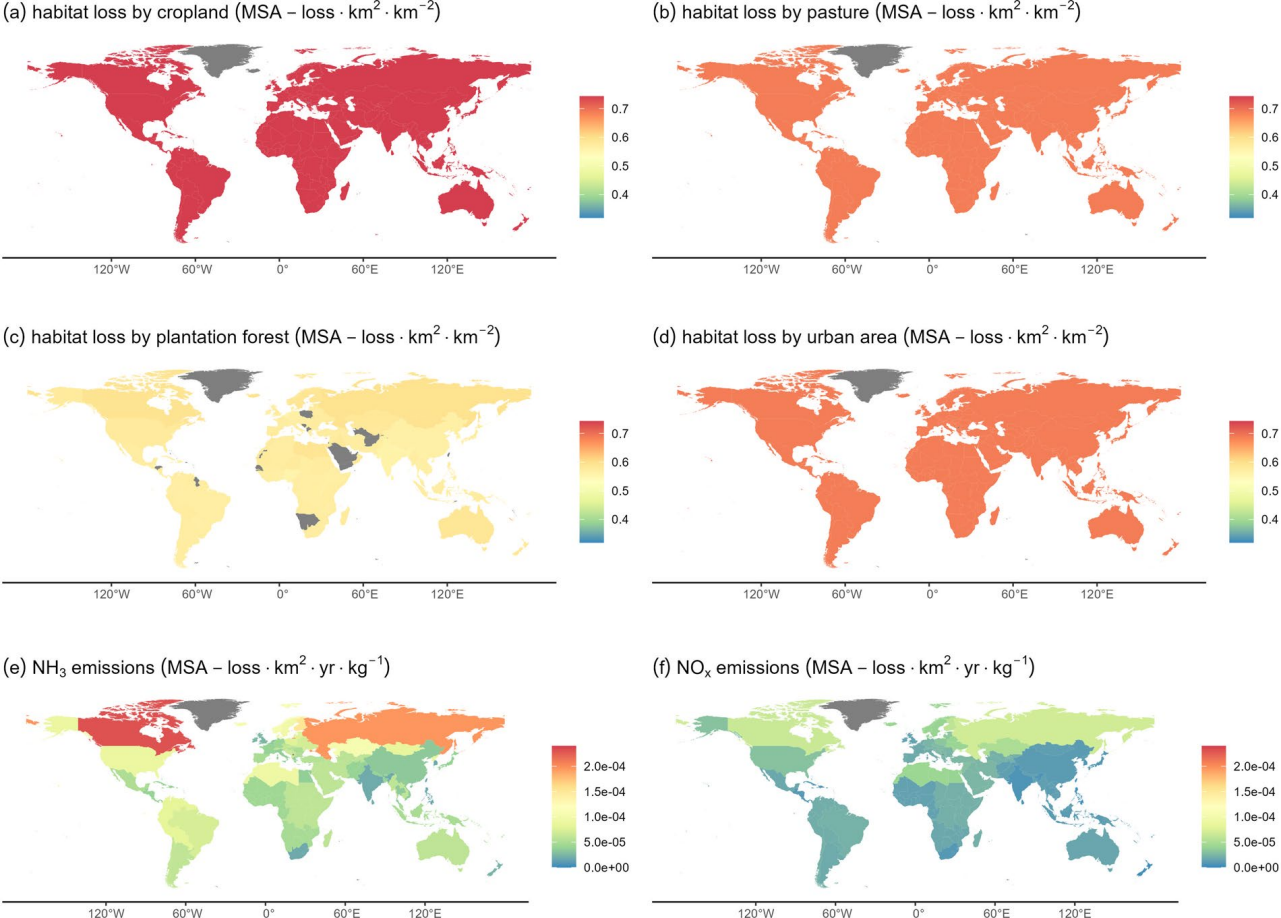
Biodiversity impact factors for plants for habitat loss due to land use (**a**–**d**) and for nitrogen emissions (**e**,**f**). Impact factors for climate change and habitat loss due to mining are not shown because they are constant. Grey denotes that, according to our data, the pressure of concern is absent in the respective country (i.e., the BIF is zero).

**Fig. 3:**
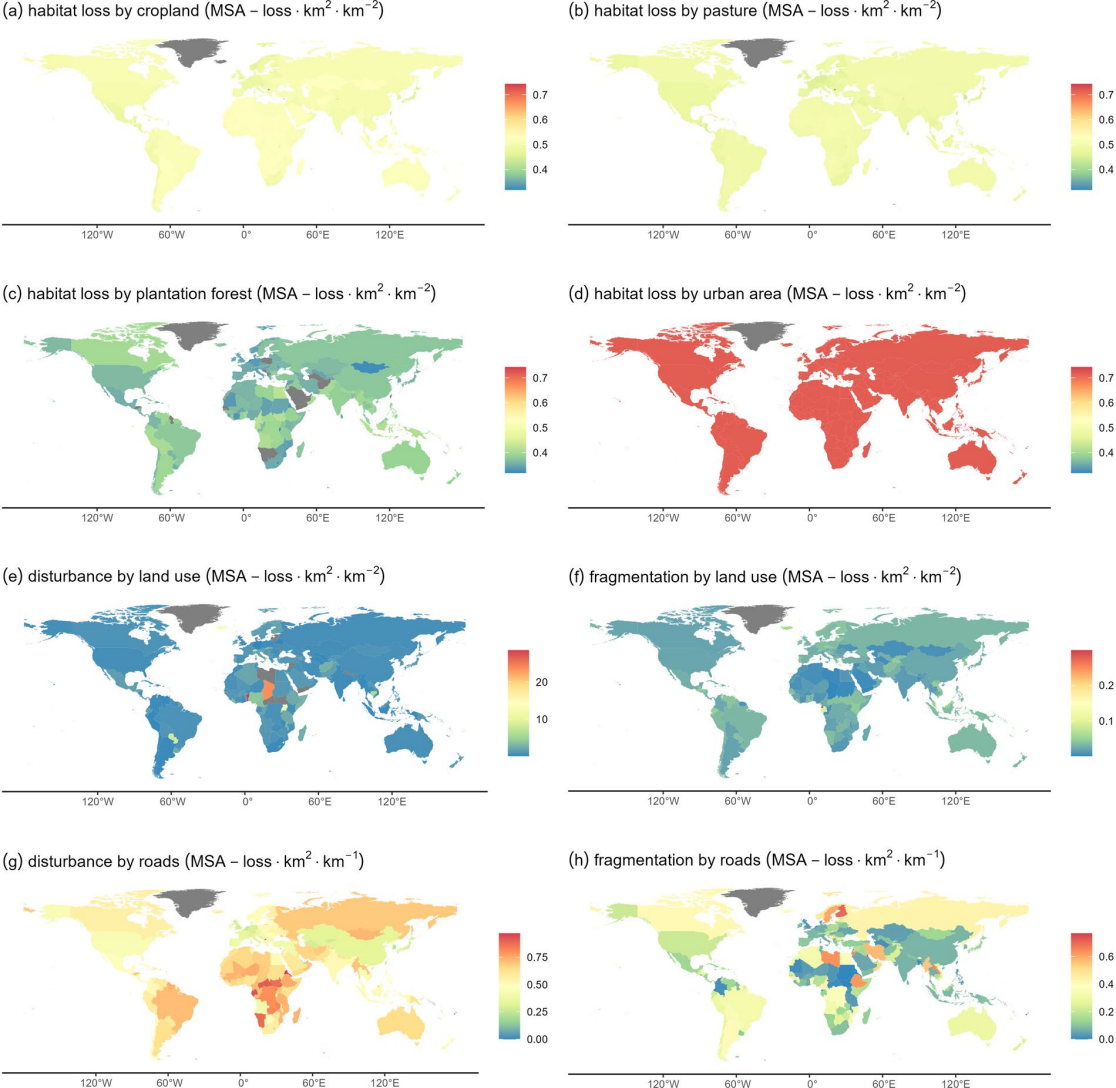
Biodiversity impact factors for warm-blooded vertebrates (birds & mammals) for habitat loss due to land use (**a**–**d**), habitat disturbance and habitat fragmentation by land use (**e**,**f**), and habitat disturbance and habitat fragmentation by roads (**g**,**h**). Impact factors for climate change and habitat loss due to mining are not shown as they are constant. Grey denotes that, according to our data, the pressure of concern is absent in the respective country (i.e., the BIF is zero).

IBIF (version 2) is available via Zenodo^36^. The dataset contains country-level BIFs for emissions of CO_2_, emissions of nitrogen (NH_3_ and NO_x_), land use (five categories) and roads, representing losses in local community intactness of plants and/or warm-blooded vertebrates (birds & mammals) corresponding with a unit of pressure generated within the country of concern. BIFs are provided per pressure and, where relevant, also per direct driver associated with the pressure (Table 2, Figs. 2, 3). For example, for roads, IBIF contains separate BIFs for the impacts of habitat fragmentation and for the impacts of habitat disturbance, in addition to an overall BIF for roads including both impacts. These separate BIFs are added to facilitate assessments of the relative contributions of different direct drivers. Further, in addition to the BIFs for each of the two species groups separately, IBIF contains overall BIFs calculated as arithmetic means of the BIFs of both groups. To calculate the arithmetic mean BIFs, we assumed a BIF of zero if, according to the assumptions in the GLOBIO model, a species group is not affected by the direct driver or pressure of concern. For example, GLOBIO assumes that warm-blooded vertebrates are not affected by nitrogen deposition, hence a BIF of zero is assumed for warm-blooded vertebrates in the calculation of the arithmetic mean BIFs for nitrogen emissions.

**Table 2:** Overview of biodiversity impact factors included in IBIF.

Pressure	Direct driver	Species group affected	Biodiversity impact factor (unit)	Typical inventory data (unit)
CO_2_ emissions	Climate change	Plants and birds & mammals	Time- and area-integrated loss of MSA per kg of CO_2_ or CO_2_ equivalents emitted (MSA-loss · km^2^ · yr · kg^−1^ CO_2_)	Emissions of CO_2_ or CO_2_ equivalents (kg)
NH_3_ emissions	Nitrogen deposition	Plants	Time- and area-integrated loss of MSA per kg of NH_3_ emitted (MSA-loss · km^2^ · yr · kg^−1^ NH_3_)	Emissions of NH_3_ (kg)
NO_x_ emissions		Plants	Time- and area-integrated loss of MSA per kg of NO_x_ emitted (MSA-loss · km^2^ · yr · kg^−1^ NO_x_)	Emissions of NO_x_ (kg)
Land use	Habitat loss	Plants and birds & mammals	Area-integrated loss of MSA per km^2^ of land converted for human use (MSA-loss · km^2^ · km^−2^)	Duration of use of cropland, pasture, plantation forest, urban land and mines (km^2^ · yr)
	Habitat fragmentation	Birds & mammals		Duration of use of cropland, pasture and urban land (km^2^ · yr)
	Habitat disturbance	Birds & mammals		Duration of use of mines (km^2^ · yr)
Roads	Habitat fragmentation	Birds & mammals	Area-integrated loss of MSA per km of road (MSA-loss · km^2^ · km^−1^)	Fuel use per ton-km of freight transport (MJ ∙ ton^−1^ ∙ km^−1^) or per person-km of people transport (MJ ∙ person^−1^ ∙ km^−1^); annual fuel use per industrial sector or by households (final demand) (MJ ∙ yr^−1^)
	Habitat disturbance	Birds & mammals		

IBIF provides impact factors for each pressure and, in addition, for corresponding direct drivers in case there are multiple direct drivers associated with a pressure. The table further shows the species groups affected per direct driver and the typical inventory data available for footprint analyses.

## Technical Validation

**Fig. 4:**
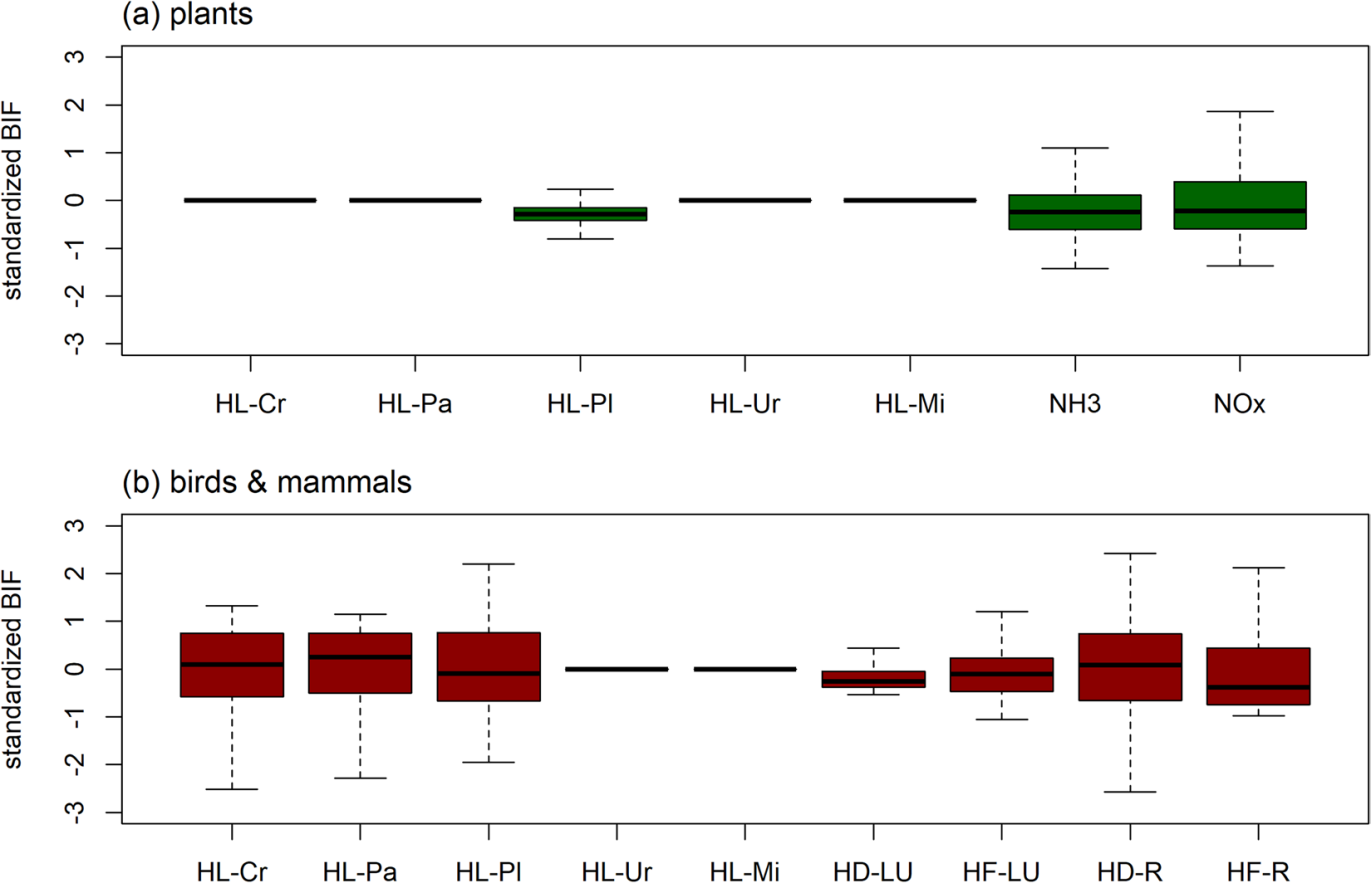
Distribution of standardized BIFs (zero mean, unit variance) across the 234 countries included in IBIF for (**a**) plants and (**b**) birds & mammals. BIFs are shown for habitat loss (HL) due to land use (including Cr = cropland, Pa = Pasture, Pl = Plantation forest, Ur = urban land, Mi = mines), NH_3_ emissions, NO_x_ emissions, habitat disturbance (HD) due to land use (LU; mines), habitat fragmentation (HF) due to land use, and habitat disturbance and habitat fragmentation due to roads (R). Boxplots show medians (horizontal black lines), interquartile ranges (25–75%; boxes) and 1.5 times the interquartile range (whiskers). BIF values beyond the whiskers are not shown.

We checked the implementation of the BIFs calculations by verifying whether the resulting output complies with the assumptions made within the GLOBIO model. For plants, GLOBIO assumes that nitrogen deposition affects only natural vegetation, semi-natural vegetation and forest plantations, and does not have an impact in urban land, cropland and pastures^19^. Hence, plant communities in urban land, cropland and pastures are assumed to be subject only to impacts of climate change and habitat loss due to land use. Because the GLOBIO model assumes impacts of a given increase in global mean temperature to be the same across the globe, BIFs for plants for these land-use types should also be constant across the globe, which is indeed the case (Fig. 4). Similarly, vertebrates in urban land are assumed to be affected only by habitat loss and climate change, resulting in a globally constant BIF for habitat loss due to urban land (Fig. 4). In other land-use types, BIFs for habitat loss are expected to show spatial variability due to the influences of other pressures (nitrogen deposition in forest plantations for plants; road disturbance in cropland, pasture and forest plantations for vertebrates; habitat fragmentation in forest plantations for vertebrates^19^), which we indeed observed (Fig. 4).

Spatial variability in nitrogen BIFs results from three elements: GLOBIO’s non-linear response relationship for nitrogen deposition (Table 1), leading to steeper declines in plant MSA at lower levels of deposition, the assumption that nitrogen deposition affects primarily natural vegetation, and the SRMs from TM5-FASST. Combined, these elements explain why we obtained high nitrogen emission BIFs for Canada and Russia (Fig. 2), which are characterized by relatively low levels of deposition hence a steep decline in MSA per kg of nitrogen deposited, large shares of natural areas, and a large share of the ammonia emissions deposited within their own territorial borders (70% and 75% of NH_3_, respectively, according to the SRM from TM5-FASST^34^). In contrast, BIFs for nitrogen are considerably lower in western European countries and in China, reflecting that these countries are characterized by higher levels of deposition hence a lower impact per kg, as well as smaller shares of natural vegetation, where nitrogen can have an impact.

We obtained high BIFs for habitat fragmentation or habitat disturbance for countries with a large share of natural vegetation and where the pressures underlying fragmentation or disturbance (urban land, cropland, mines and roads) are relatively small. In these cases, the area-integrated impacts of fragmentation or disturbance are assigned to a small area of land used as cropland, pasture, urban land or mines or a small total road length, resulting in a relatively large impact per unit of land-use area or road length. For example, the northern part of Chad is characterized by vast areas unaffected by land use other than mining, resulting in a large area being affected by disturbance by mines hence a large BIF.

## Usage Notes

### Quantifying biodiversity footprints with IBIF

The impact factors in IBIF^36^ can be used to quantify the biodiversity footprints of products, industrial sectors or consumers. This can be done for vascular plants and warm-blooded vertebrates (birds & mammals) separately or for both groups combined. For product footprints, the BIFs can be combined with so-called life cycle inventory (LCI) data, representing the environmental pressures (emissions and resource uses) associated with a given economic activity. To quantify the biodiversity footprints of industrial sectors or consumers, the BIFs can be combined with environmentally extended multi-regional input-output (EEMRIO) data, which combine data on trade flows between different industrial sectors and regions with information on the resource use and emissions of each industrial sector in each region^13^. BIFs for CO_2_ emissions, nitrogen emissions and land use are readily linked to LCI data or environmental extensions in MRIO models, as their units match (Table 2). Specifically, emissions expressed in kg of CO_2_, NH_3_ or NO_x_ can be directly connected to the corresponding BIFs, and the same holds for land use activities expressed in km^2^ · yr. For greenhouse gasses other than CO_2_, a user could convert the BIFs based on the differences in global warming potential between the greenhouse gas of concern and CO_2_ (Table 3). We also stress here that the BIFs for CO_2_ emissions are based on a time horizon of 100 years, and that a shorter or longer time horizon would result in smaller or larger BIFs and resulting footprints, respectively. Should a user wish to adopt a different time horizon, they could implement a correction factor based on the differences in integrated absolute global temperature change potential (IAGTP) between their desired time horizon and our default (Table 3).

**Table 3:** Conversion factors for BIFs for CO_2_ as included in IBIF^36^.

Greenhouse gas	Time horizon (years)		
	20	100	500
CO_2_	0.190	1	4.71
CH_4_ (fossil)	15.7	29.8	47.1
CH_4_ (non-fossil)	15.1	27.0	33.9
N_2_O	51.8	273	612

In IBIF, the default BIF for CO_2_ emissions is based on a time horizon of 100 years^13,33^. By multiplying the default CO_2_ BIF with a conversion factor from this table, a user can adopt a different time horizon and/or quantify greenhouse gas footprints of other common greenhouse gases. Conversion factors were derived from the integrated global warming potentials of CO_2_ as reported by Joos *et al*.^33^ combined with global warming potentials (GWPs) obtained from Forster *et al*.^38^.

Road impacts, in IBIF quantified as MSA-loss ∙ km^2^ per km of road, can to be attributed to economic actors (sectors or consumers) or activities proportional to their use of roads. EEMRIO data typically include information on fuel use per actor for the purpose of transport, which can be used as a basis to attribute road impacts^13^, as:

7 $${BF}_{R,m,r}={BIF}_{R,r}\cdot {RL}_{r}\cdot T\cdot \frac{{FU}_{m,r}}{{FU}_{r}}$$

where *BF*_*R,a,r*_ represents the direct biodiversity losses due to roads assigned to actor *m* (i.e., an industrial sector or the consumers) in region *r* in a specific year (MSA-loss · km^2^ · yr), BIF_R,r_ represents the BIF for road impacts in region *r* as included in IBIF (MSA-loss · km^2^ · km^−1^), RL_r_ is the total road length (km) in region *r*, T is the duration of road use (1 year), FU_m,r_ is the direct fuel use of actor *m* in region *r* specifically for transport (MJ · yr^−1^), and FU_r_ is the total direct fuel use for transport across all sectors and consumers in region *r* (MJ · yr^−1^).

For LCA applications, the BIFs for roads need to be converted from biodiversity impacts per km of road to impacts either per ton-km road transport (for transport of goods) or per person-km road transport (for transport of people). This can be achieved as follows:

8 $${{BIF}}_{R,a,r}={{BIF}}_{R,r}\cdot {{RL}}_{r}\cdot \frac{{{FU}}_{a,r}}{{{FU}}_{r}}$$

where *BIF*_*R,a,r*_ represents the biodiversity impact factor for roads for activity *a* in region *r* (MSA-loss · km^2^ · yr · ton^−1^ · km^−1^ for transport of goods or MSA-loss · km^2^ · yr · person^−1^ · km^−1^ for transport of persons), and FU_a,r_ is the direct fuel use per person-km or ton-km for activity *a* in region *r*, as available in LCI databases (MJ · ton^−1^ · km^−1^ or MJ · person^−1^ · km^−1^).

Data on road length (RL_r_) can be obtained from the GRIP database and accompanying paper, which contain a shapefile with roads as well as a table with the total road length per country^30^. To ensure consistency with the BIFs, we recommend to use only road types 1–3. Data on fuel use of sectors and consumers (households) (FU_a,r_ and FU_r_) can be obtained from, for example, the OECD (https://www.oecd.org/en/data.html), while data on the fuel use of activities (FU_a,r_) is available from the ODYSSEE-MURE portal (https://www.indicators.odyssee-mure.eu/key-indicators.html).

## Limitations

With respect to the applicability of the impact factors in IBIF, we emphasize here that we obtained the factors through GLOBIO model simulations with input data on climate change, atmospheric nitrogen deposition and land use representative of the year 2020. This implies that the BIFs can be used to quantify the biodiversity footprints of current products, sectors or consumers, yet are not necessarily applicable for assessments of historic or prospective footprints, given that pressure impacts on MSA are contingent on the magnitude of that pressure as well as the influences of other pressures. This, in turn, implies that a proper assessment of historic or future footprints requires re-running GLOBIO with historic or potential future pressure input data in order to derive a representative set of BIFs. We also stress that the time-integration in our BIFs hence resulting footprints represents the duration of the change in state of the environment and the biodiversity loss corresponding with this change in state, without consideration of the time required for biodiversity to recover once the pressure is released (for example, upon land abandonment). Further work is needed to integrate the time required for biodiversity to recover upon release of a pressure into the BIFs included in IBIF.

It is important to keep in mind that the current version of GLOBIO uses universal MSA impact relationships (Table 1), which implies that, all other pressures being equal, a loss in MSA due to a given pressure is the same everywhere. Hence, biodiversity footprints quantified based on IBIF do not account for spatial differences and gradients in community composition and species richness across the globe. For a more comprehensive assessment of footprints, we therefore recommend to use multiple complementary biodiversity metrics, including at least one metric that is indicative of (endemic) species loss in addition to the local intactness-based metric used here^37^. Finally, we note that our BIFs are associated with uncertainty due to, among others, the uncertainty in the MSA impact relationships in GLOBIO resulting from the heterogeneity in the underlying data^19^. Further work remains to quantify the corresponding uncertainty in the BIFs through probabilistic simulations, which was not feasible at the time of this study in view of the computational intensity of the GLOBIO simulations.

## Data Availability

The GLOBIO model code used to obtain the MSA losses (Eq. 1) and convert these into BIFs (GLOBIO v4.3.1) is available at https://github.com/GLOBIO4/GlobioModelPublic.
